# The Development of Evidence-Based Classification of Vision Impairment in Judo: A Delphi Study

**DOI:** 10.3389/fpsyg.2019.00098

**Published:** 2019-02-15

**Authors:** Kai J. Krabben, Rianne H.J.C. Ravensbergen, Hiroki Nakamoto, David L. Mann

**Affiliations:** ^1^Department of Human Movement Sciences, Faculty of Behaviour and Movement Sciences, Vrije Universiteit Amsterdam, Amsterdam Movement Sciences, Amsterdam, Netherlands; ^2^Faculty of Physical Education, National Institute of Fitness and Sports in Kanoya, Kanoya, Japan

**Keywords:** Paralympic, classification, judo, vision impairment, Delphi

## Abstract

**Objective:** Most para-sports group athletes into “classes” to compete against others with similar activity limitations. Judokas with vision impairment (VI) instead all compete in the same class irrespective of their level of impairment. There is considerable controversy whether this approach represents a legitimate way to structure judo competition. The aim of this study was to establish expert opinion on the requirements for an evidence-based classification system for VI judo.

**Methods:** A panel of 18 athletes, coaches, and administrators participated in a three-round Delphi review process. Expert opinions were canvased for a large range of issues related to classification in judo. Between rounds, results were summarized and further questions were asked on topics where consensus was not reached across experts.

**Results:** The panel expressed that: (i) blind and partially sighted athletes should not compete against each other in the same class; (ii) additional measures of visual function might be needed to accurately evaluate an athlete’s impairment; (iii) the minimum impairment criteria (MIC) should represent a more severe level of impairment to ensure that all those included possess a level of VI that indeed decreases performance in judo; and (iv) legitimate competition could be undermined by some athletes intentionally underperforming on classification tests. The panel identified six additional measures of visual function which are not currently measured but are likely to impact judo performance, and six aspects of judo performance which are most likely impacted by VI.

**Conclusion:** Experts in the field of VI judo expressed a need to change the manner in which VI judokas are classified. This study outlines a model for establishing the impairment–performance relationship and guides the development of evidence-based classification for VI judo.

## Introduction

To ensure Paralympic medals are awarded to the best athletes and not simply to those who are least impaired, para-sport competitions are divided into “classes” that are defined based on the type and degree of an athlete’s impairment. The process of grouping para-athletes together for competition on the basis of their impairment is known as *classification.* The aim of classification is to minimize the impact of impairment on the outcome of competition (Tweedy and Vanlandewijck, 2011). Classification in para-sports is a two-step process. First, a decision is made whether an athlete’s impairment is severe enough to warrant eligibility to compete. Thus, minimum impairment criteria (MIC) are established, which by definition describe the level of impairment that has an impact on performance in that sport (International Paralympic Committee, 2016)^1^. If an athlete is eligible to compete, the second step is to allocate a *sport class* to the athlete to compete against others whose impairments result in a similar activity limitation (International Paralympic Committee, 2015).

The *IPC Athlete Classification Code* (International Paralympic Committee, 2015) requires all Paralympic sports to adopt an *evidence-based* classification system that is developed using evidence which demonstrates the relationship between impairment and performance in that sport. Consequently, classification also needs to be *sport-specific*, because the impact of an impairment is likely to differ depending on the unique demands of each sport (i.e., a leg amputation impacts performance in running differently than in swimming). Following the formulation of the Athlete Classification Code, the IPC adopted a Position Stand describing how classification research should be conducted (Tweedy and Vanlandewijck, 2011). The basic approach is to correlate measures of functional impairment with measures of sport-specific performance. Once the impairment–performance relationship has been established, classes can then be formed for athletes with a similar activity limitation within the sport.

While the move toward sport-specific classification has progressed in sports that cater for athletes with physical and intellectual impairment, most sports for athletes with vision impairment (VI) continue to use a long-standing system that employs the same class structure irrespective of the sport. VI athletes are currently allocated to one of three different sport classes (B3, B2, or B1^2^) that differ according to the severity of impairment. These criteria were developed on the basis of the World Health Organization’s definitions of low vision and blindness (World Health Organization, 2004). The current system was recently evaluated by a panel of experts across thirteen different VI sports (Ravensbergen et al., 2016). These experts reached consensus that the existing VI classification procedures do not achieve the aim of minimizing the impact of impairment on performance, with a lack of sport-specificity being the main limitation. Subsequently, a VI-specific Position Stand has been formulated and adopted by both the IPC and the International Blind Sports Federation (IBSA). This *VI position stand* outlines suitable approaches for conducting research into the impairment–performance relationship in VI sports (Mann and Ravensbergen, 2018). In agreement with the expert consultation, the VI position stand highlights the need for sport-specificity in classification research. Key sport-specific decisions that need to be made are (i) which aspects of vision should be measured, (ii) how performance should best be measured in that sport, and (iii) under what conditions vision testing should take place.

VI judo is a Paralympic sport where particular controversy exists around the way that athletes are classified. Even though athletes are allocated one of the three classes (B3, B2, and B1), as they are in most other VI sports, during competition all athletes compete against each other regardless of their allocated class. Effectively VI judo thus has only one sport class, with all athletes competing against each other irrespective of whether they are partially sighted or completely blind. Blind athletes are afforded no handicapping or advantage during competition, with the classes only used for the purposes of allocating award ranking points for the World Ranking List on the basis of the match outcomes^3^. The rationale behind this historical choice to have a single competition in which athletes from all classes compete is that the adaptations to the competition rules in VI judo increase the likelihood that those with more impairment are not disadvantaged when competing against others with less impairment (Krabben et al., 2018). In the able-sighted equivalent, athletes commence their bout 4 m apart and must first compete to obtain an advantageous grip on their opponent before battling while in-contact. Because of the presumably high visual demands of this *grip fighting* (Piras et al., 2014), athletes in VI judo instead start with a grip of their opponent already in place. This adaptation presumably lowers the visual demands of the sport, making it more suitable for those with VI by providing an opportunity to rely largely on other sources of information (e.g., haptic, kinesthetic). However, it is doubtful whether the rule adaptations are sufficient to allow judokas with different degrees of VI to compete equitably against each other. Recent analyses of competition outcomes suggest that under the present system, blind judokas might be at a disadvantage when competing against partially sighted opponents (Krabben et al., 2018; Mashkovskiy et al., 2018). Yet no studies have been carried out which directly relate measures of functional vision to judo performance. This information is required to determine how many classes are warranted for VI judo and where the boundaries between classes should be set (Tweedy and Vanlandewijck, 2011).

A critical barrier to the design of experimental studies that investigate the impairment–performance relationship in VI judo is the lack of theoretical understanding of the visual demands of judo. Also, a theoretical model of the key determinants of judo performance affected by vision loss is required to understand the best approach(es) to investigate the impairment–performance relationship in the sport (Tweedy et al., 2016). The VI position stand (Mann and Ravensbergen, 2018) suggests that the first step in this process should be to consult with experts in a sport (i.e., athletes, coaches, administrators) to determine the measures of visual function and performance that should be chosen to model the impairment–performance relationship in a given VI sport, and under what circumstances these measures should be collected. For instance, only visual acuity and visual field are currently assessed during VI classification, yet there may be other aspects of visual function which are more important for judo (e.g., the ability to adequately perceive motion or depth might be crucial to successfully anticipate and react to an opponent’s attack). Moreover, performance on these tests could be evaluated under different conditions, e.g., using one or both eyes, and with or without optical correction. The best way of testing vision is likely to depend on the nature of the sport, for instance, on the ability of athletes to wear optical correction during training or competition (Ravensbergen et al., 2016). Sport-specific knowledge is most of all required to understand what aspects of judo performance are most likely to be impacted by VI. Within an interactive, skill-based sport such as judo, the outcome of a match is likely to rely on an interplay of many different factors. Expert knowledge is helpful to decide what aspects of performance are mostly visually guided, and therefore would be worthwhile to measure in classification research. Expert consultation thus functions to identify a candidate list of both impairment and performance measures, which can then be tested empirically to quantify their associative strength (Mann and Ravensbergen, 2018). Expert opinion might also be desirable to make other sport-specific decisions for issues on classification research in judo, such as whether or not to account for the age at which VI was acquired (Ravensbergen et al., 2016).

The aim of this study was to establish expert opinion on the requirements for a sport-specific system of classification for VI judo. We consulted with experts to evaluate to what extent the current classification system in VI judo provides fair competition for all athletes, and to decide (i) which aspects of vision are most likely to impact judo performance, (ii) which aspects of judo performance are most likely to be impacted by VI, (iii) under what conditions vision testing for judo classification should take place, and (iv) other issues specific to classification research in VI judo.

## Materials and Methods

In this study, we applied the Delphi technique, which offers a structured method to evaluate the opinion of a panel of experts (Linstone and Turoff, 1975; Hasson et al., 2000; Jorm, 2015). During the Delphi process, experts respond to a series of surveys. Following each survey, experts receive feedback summarizing the panel’s previous answers before new questions are posed.

### Participants

Eighteen participants (M ± SD = 42.6 ± 12.2 years, three female) took part in the expert panel. Appropriate panelists were identified with the assistance of the IBSA Judo Committee. Participants were required to possess expertise in VI judo at an international level in their role as either an athlete (current or former), coach and/or administrator (see Table 1). They also needed to be proficient in English or have access to a translator to assist in responding to the surveys. Nineteen candidate panelists were approached to take part. All but one accepted the invitation and were included in the expert panel. The study was approved by the Scientific and Ethical Review Board of the Faculty of Behavioural and Movement Sciences at the Vrije Universiteit Amsterdam. All participants provided electronic informed consent to take part.

**Table 1:** Participant characteristics.

	*N* (%)
**Sex**	
Male	15 (83%)
Female	3 (17%)
**Continent**	
Asia	2 (11%)
Europe	11 (61%)
North America	4 (22%)
South America	1 (6%)
**Role within VI judo**^∗^	
Administrator	3 (17%)
Athlete	8 (44%)
Coach	7 (39%)
Classifier	2 (11%)
Referee	3 (17%)
**Years of experience in VI sport within each role**	
0–10	6 (33%)
10–20	8 (44%)
>20	4 (22%)


*Asterisk (^∗^) indicates that more than one answer was possible. Percentages are based on the number of individuals, not answers*.

### Procedure

The study ultimately consisted of three rounds of web-based surveys (Qualtrics Research Suite, Qualtrics, Provo, UT, United States) that covered a range of topics specifically related to evidence-based classification in VI judo (using 10 sections, see Table 2). The initial survey aimed to discuss current procedures in VI judo classification and address our main aim for the development of sport-specific classification in VI judo. The topics covered follow both the recommendations from the VI position stand and specific issues to VI judo (e.g., impact of VI across weight categories). The initial survey posed largely open-ended questions, with the second and third rounds posing largely closed questions based on the answers provided in the previous round(s). Each section of the survey started with a short summary of background information concerning the topic and/or a summary of previous responses and comments. To ensure that no relevant topics were overlooked, we provided panelists the opportunity at the end of each survey to raise any additional issues they felt were important but had not yet been addressed in the survey.

**Table 2:** Description of the 10 different sections covered throughout the surveys, along with the central question each section aimed to investigate.

	Title	Central question
Section 1	Aim of classification	Does the current system of classification within VI judo achieve its aim of minimizing the impact of impairment on the outcome of competition?
Section 2	Minimum impairment criteria	Are the current minimum impairment criteria for VI judo set appropriately or should they change, and if so how?
Section 3	Sport classes	Is it appropriate that all eligible athletes in VI judo compete against each other, or should additional sport classes be created?
Section 4	Measures of visual function used during classification	Which aspects of vision are most likely to impact on judo performance?
Section 5	Impact of vision impairment on different aspects of performance	Which aspects of performance are most likely to be impacted by vision impairment?
Section 6	Vision testing conditions	Under what conditions should vision testing in classification for VI judo take place?
Section 7	Impact of VI across different weight categories	Does the impact of vision impairment on judo performance differ across weight categories?
Section 8	Impact of a congenital compared to an acquired impairment	Does the impact of vision impairment on judo performance differ between athletes with congenital and acquired impairments?
Section 9	The use of blindfolds	Would the use of blindfolds be an appropriate way to ensure fair competition within VI judo?
Section 10	Intentional misrepresentation	Is the legitimacy of VI judo considered to be under the threat of athletes deliberately underperforming on classification tests?


During the closed questioning, statements were posed to panelists who typically had to state whether they agreed or disagreed with the statement. In these cases, the aim was to reach consensus, defined as a threshold of at least 75% agreement across panelists calculated separately for each question. This threshold is conservative when compared to other Delphi studies (Hasson et al., 2000 report values between 51 and 80%). Because of the technical nature of some questions, panelists were provided with the option to respond that they did not feel qualified to answer that question. In those cases, their response was excluded from the calculation for 75% agreement (see Supplementary Table S1). Moreover, panelists always had the opportunity to elaborate on their answer. A summary of these comments was provided as feedback to all panelists in the following round, and those comments were used to re-phrase statements or pose additional questions where necessary in the following survey. Between rounds, responses were summarized and discussed between the authors. A follow-up survey was then drafted, which was again discussed between all authors before being finalized and sent to the panelists. Topics on which consensus was reached were no longer discussed in further surveys. Topics where consensus was not yet reached, were clarified and/or rephrased based on the comments made by the panelists (see Supplementary Table S2). All three rounds were completed over a period of 8 months. No statistical analyses were applied for this study beyond the calculation of simple percentages.

## Results

The first and second surveys were completed by all 18 panelists, and 17 completed the final survey^4^. We now discuss the key results for each 10 section addressed in the surveys (for all final results, see Supplementary Table S1; for the process of reaching consensus within each section, see Supplementary Table S2).

### Section 1: Aim of Classification

The aim of Paralympic classification is *to minimize the impact of eligible impairments on the outcome of competition*. The panel reached consensus (83%) that this aim is not entirely fulfilled in VI judo using the existing classification system (61% no; 22% partially; 17% yes). Panelists most frequently commented that: (i) it is unfair that blind athletes should compete against partially sighted athletes; (ii) the current assessment of vision during classification does not accurately capture the functional impact of VI on judo performance; and (iii) fair competition is presently being undermined by the number of athletes believed to be intentionally underperforming on classification tests. We elaborate on these concerns in the sections below.

### Section 2: Minimum Impairment Criteria

The existing classification system considers an athlete to be eligible to compete in VI judo when their visual acuity^5^ is equal to or worse than 1.0 logMAR, or when the diameter of their visual field^6^ is no more than 40°. The panel unanimously agreed (100%) that the MIC for VI judo should not become more inclusive such that it would include athletes whose vision is *better* than the present MIC. Panelists felt the MIC should either be less inclusive (i.e., excluding some athletes who are currently eligible to compete, 62%), or that they should remain unchanged (38%). The panel agreed (80%) that further research is needed to provide an evidence-based definition of the MIC for judo. Panelists also reached consensus (83%) that athletes who meet the MIC for VI judo should also be allowed to compete in able-sighted judo competition. This view is consistent with the current regulations.

### Section 3: Sport Classes

VI judo effectively offers only one sports class, with all eligible athletes competing against each other. The panel agreed (78%) that splitting VI judo into more than one sport class would increase the fairness of competition. The key concern raised by the panel was that blind and partially sighted athletes currently need to compete against each other. We therefore posed a follow-up question asking specifically whether a separate class for *blind* judokas would improve the fairness of competition. The panel indeed reached consensus (78%) that a separate class for blind athletes would be fairer than the present competition format. Reasons included that: (i) although VI judo starts with the grip in place, the rules allow athletes to (briefly) release one hand and re-grip (and some vision would assist in doing so); (ii) the ability to see *some* movement provides a strong performance advantage even once a grip is in place (e.g., for seeing an opponent’s legs); (iii) blind judokas have a disadvantage in acquiring sport-specific skills, because they cannot make use of observational learning by seeing a technique being demonstrated to them; and (iv) blind athletes are unable to study video footage of their opponents or observe them during competition to learn about their patterns of play.

There is a restriction in the number of medal events at the Paralympic Games. If there were to be an increase in the number of sport classes for VI judo, but no change to the number of medal events, then events would be held at the Paralympic Games for only some of the sport classes in judo (i.e., some athletes might no longer be able to compete at the games). For VI judo, there are presently 13 medal events (seven weight classes for men and six for women) at the Paralympic Games. This means that if there were to be an increase in the number of classes for VI judo, it might not be possible to simply hold a medal event for each of the new VI classes in each of the weight categories. We therefore asked the panel for proposals on how to structure competition to deal with this restriction, if there were to be a recommendation to increase the number of sport classes. Two different proposals were raised as a result of open-questioning in the first survey, both of which we elaborated on in subsequent surveys. In Option 1, some panelists proposed that this issue could be overcome by reducing the number of weight classes, for instance by adopting light-, middle-, and heavyweight classes. In Option 2, it was proposed that all current weight categories could remain, but that only blind athletes should compete in the Paralympic Games (as is currently the case in VI football 5-a-side). The panel did not reach consensus whether they preferred Option 1, Option 2, or the present competition format (i.e., with all athletes competing within the same class): 31% preferred Option 1, 25% preferred Option 2, and 44% favored the current system. Panelists reported a variety of reasons for preferring one proposal over the other. Some panelists disliked Option 1 because they prefer the structure of VI judo and able-sighted judo to remain similar, or because they doubted that athletes from different weight categories are able to compete against each other equitably. Others opposed Option 2 because it would lead to a decrease in competitive depth, as they believed this would decrease the number of participating athletes within a class. In conclusion, although our panel clearly identified a need to change the current system (see Section 1), they could not readily agree how this change should be achieved.

### Section 4: Measures of Visual Function Used During Classification

Classification in VI judo is currently performed measuring only visual acuity and visual field, with recent work demonstrating support across all VI sports for the inclusion of additional measures during classification (Ravensbergen et al., 2016). The panel could not agree whether visual acuity and visual field are the *only* measures of visual function that should be used for classification in VI judo (42% yes, 58% no). In a follow-up question, we asked the panel to indicate which of the visual functions on a list^7^, they considered important enough for judo performance to be included in classification. Results are presented in Table 3. The panel unanimously agreed that both visual acuity and visual field are important enough to be included in classification. Panelists commented that these are globally standardized measures that impact many aspects of our use of vision in daily life as well as during training or competition. A majority, however, without reaching consensus, also believed that other measures such as motion perception, dynamic visual acuity, and light sensitivity should be included in classification. The main comment was that classification research should consider many possibly relevant aspects of VI to fully understand its impact on judo performance. In conclusion, there is clear support for the continued use of visual acuity and visual field during classification, and some limited support for the consideration of other measures of vision.

**Table 3:** List of additional measures of vision impairment considered by the panel for inclusion in classification.

Measure of vision	Description	% Agreement that measure is important enough to include in classification
Visual acuity	A measure of the sharpness/clarity of vision.	100%
Visual field	A measure of the area of peripheral vision with which an individual can see (i.e., without moving their eyes)	100%
Motion perception	The ability to estimate the speed and the direction of a moving object	71%
Dynamic visual acuity	A measure of the sharpness/clarity of vision when observing a moving target	69%
Light sensitivity	The impact of bright lights on the ability to see clearly	69%
Ocular coordination	The ability of both eyes to move together in cooperative fashion	64%
Depth perception	The ability to perceive the world in three dimensions, e.g., to estimate the distance to an object	56%
Contrast sensitivity	The ability to distinguish objects from a background	53%
Color vision	The ability to distinguish different colors	19%


*Panelists were asked to rate which measures they believed to be important enough to be included in classification*.

### Section 5: Impact of Vision Impairment on Judo Performance

**FIGURE 1:**
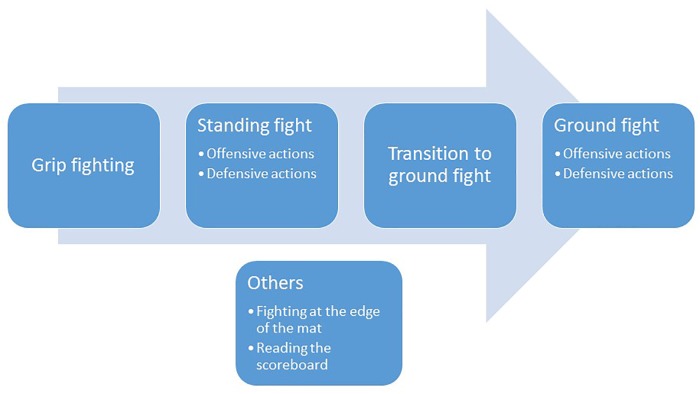
Model of different aspects of judo performance in which vision plays a role. Judo starts with a *grip fighting* phase, in which athletes attempt to obtain an advantageous grip over their opponent. They then proceed to the *standing fight*, in which athletes try to unbalance and throw their opponent (*offensive actions*), while avoiding being thrown (*defensive actions*). After a throw, a judo contest continues as a *ground fight*. During the *transition* from standing to ground fight, an athlete must adapt quickly to start the ground fight in an advantageous position. During the ground fight, the athlete must aim to score by pinning or submitting the opponent (*offensive actions*) and/or must prevent their opponent from scoring (*defensive actions*). If no progress in the ground fight is made within a reasonable time period, the referee pauses the match and the athletes stand to continue the match from their starting positions. During the breaks in the fight, athletes may want to *read the scoreboard* to check on the score and time left on the clock. The final aspect in the model is the ability to *fight at the edge of the mat*, which is important during all phases of the game but especially during the grip fight and the standing fight. Because athletes are penalized for stepping out of the mat area, it offers a tactical advantage to trap the opponent at the edge of the mat and thereby limit their freedom of movement.

A sport-specific system of classification for VI judo must be based on research demonstrating the impact of impairment on sport-specific measures of judo performance (Tweedy and Vanlandewijck, 2011). However, for judo, this is not a trivial issue. It is not immediately clear how judo performance should be measured given that competition outcome depends not only on an athlete’s own ability, but also on that of their opponent. To guide the analysis of performance in judo classification research, we asked the panelists in the initial survey to list all aspects of judo performance (either VI or able-sighted) that they thought might be negatively impacted by VI. From their suggestions, we developed a theoretical model to better understand the impact of vision loss on judo performance. This model comprises of eight aspects of judo performance in which vision may play a role (see Figure 1).

Having developed the model relating vision and performance in judo, we sought to establish (i) the relative importance of each aspect to overall performance and (ii) the importance of vision for each aspect. To address the former, we asked the panel to rate on a Likert scale how important each was for winning a match in VI judo (see Table 4). The aspects of performance rated most important for competitive success were offensive and defensive skills in the standing fight (both judged as very to extremely important by 88% of panelists). Other aspects of performance rated highly were the transition from standing to ground fight, and the defensive and offensive skills in the ground fight. Grip fighting skills were considered moderately to extremely important by 75% of the panelists; one panelist commented that even though VI judo starts with a grip in place, re-gripping to a more advantageous position still plays an important role in the sport. Fighting at the edge of the mat (i.e., trapping an opponent on the edge so that they have less space to move) and reading the scoreboard were both considered as slightly to moderately important by 75% of the panel. Some panelists commented that the rules for stepping out of the mat area are applied less strictly in VI judo compared to able-sighted judo, so therefore the tactical importance of fighting at the edge of the mat is lower.

**Table 4:** Relative importance of different aspects of judo performance.

Aspect of judo performance	Not at all important	Slightly important	Moderately important	Very important	Extremely important	Level of consensus for importance
Offensive skills in standing fight	0%	0%	13%	19%	69%	Very to extremely (88%)
Defensive skills in standing fight	0%	6%	6%	31%	56%	Very to extremely (88%)
Transition from standing to ground fight	0%	0%	19%	38%	44%	Very to extremely (81%)
Defensive skills in ground fight	0%	6%	13%	44%	38%	Very to extremely (81%)
Offensive skills in ground fight	0%	6%	19%	31%	44%	Very to extremely (75%)
Grip fighting skills	0%	25%	6%	19%	50%	Moderately to extremely (75%)
Fighting at the edge of the mat	0%	44%	31%	19%	6%	Slightly to moderately (75%)
Reading the scoreboard	25%	25%	50%	0%	0%	Slightly to moderately (75%)


*Panelists were asked to answer on a five-point Likert scale ranging from “not at all important” to “extremely important.” The level of consensus was determined by taking the smallest range of answers given by at least 75% of the panelists. The list is ordered according to the rankings of the experts from most to least important to obtain competitive success in VI judo.*

Next, we asked the panelists to rate the strength of the impact of VI on each aspect of performance during competition. Since performance in judo is always relative to an opponent, we asked the panelists to compare the relative advantage of a judoka with full vision when fighting under VI judo rules against (i) an opponent who only just meets the MIC and (ii) a fully blind opponent to establish what aspects of performance might be impacted by VI (see Table 5). Overall, the panel felt that against an opponent with full loss of vision, a fully sighted judoka would hold an advantage for each aspect of performance to some degree, including those aspects that were rated most important for winning a match in VI judo. Against an opponent with partial vision who just meets the MIC, the panel felt that the difference in vision generally offered only a mild to moderate advantage or even no advantage to the fully sighted judoka.

**Table 5:** Impact of vision impairment on different aspects of judo performance.

		No advantage	Slight advantage	Moderate advantage	Large advantage	Very large advantage	Level of consensus for advantage
Fully sighted vs just eligible	Offensive skills in standing fight	44%	25%	19%	13%	0%	None to moderate (88%)
	Defensive skills in standing fight	50%	25%	13%	13%	0%	None to slight (75%)
	Transition from standing to ground fight	31%	31%	25%	13%	0%	None to moderate (75%)
	Defensive skills in ground fight	75%	0%	19%	6%	0%	None (75%)
	Offensive skills in ground fight	69%	6%	19%	6%	0%	None to moderate (75%)
	Grip fighting skills	31%	25%	19%	25%	0%	None to moderate (75%)
	Fighting at the edge of the mat	7%	27%	27%	27%	13%	None to moderate (81%)
	Reading the scoreboard	50%	19%	13%	13%	6%	Slight to large (80%)

Fully sighted vs fully blind	Offensive skills in standing fight	13%	0%	19%	31%	38%	Moderate to very large (88%)
	Defensive skills in standing fight	19%	13%	13%	25%	31%	Slight to very large (81%)
	Transition from standing to ground fight	6%	13%	6%	31%	44%	Large to very large (75%)
	Defensive skills in ground fight	25%	13%	31%	13%	19%	Slight to very large (75%)
	Offensive skills in ground fight	13%	19%	31%	13%	25%	Slight to very large (88%)
	Grip fighting skills	6%	13%	0%	38%	44%	Large to very large (81%)
	Fighting at the edge of the mat	6%	0%	0%	6%	88%	Large to very large (80%)
	Reading the scoreboard	7%	7%	7%	20%	60%	Very large (88%)


*Panelists rated the extent to which performance is impacted for an athlete who is just eligible to compete in VI judo when fighting either an opponent who is just eligible to compete in VI judo, or a fully sighted opponent (under the rules of VI judo). Panelists were asked to answer on a five-point Likert scale ranging from “no advantage” to “very large advantage.” The level of consensus was determined by taking the smallest range of answers given by at least 75% of the panelists. The lists are ordered according to the rankings of the experts from most to least important to obtain competitive success in VI judo (see Table 4)*.

### Section 6: Vision Testing Conditions

For athletes in all VI sports, visual function is being assessed for classification while wearing the best possible optical correction (International Paralympic Committee, 2016). Each eye is then tested individually, and the athlete’s eligibility and sport class are determined on the basis of the results of their better performing eye. These procedures have been called into question by those within VI sport, because they may not provide a representation of the habitual vision of the athlete in their performance environment (Ravensbergen et al., 2016). For instance, in the case of the best optical correction, it could be that the athlete is unable to wear that correction during competition and so would compete with worse vision than that which was tested during classification. Our panel, however, agreed (76%) that the current procedure of testing VI judokas while wearing the best possible optical correction was appropriate, irrespective of whether or not the correction could be worn on the mat. The main comment was that an athlete might still benefit from using optical correction away from competition (i.e., by observing others demonstrating techniques to them in training, or to study fights of their opponents).

In competition, judokas rely on vision from both eyes combined, yet they are currently classified on the basis of the function of their best eye only. Crucially, when tested with two eyes together rather than the best eye only, the measured visual function of some athletes will improve whereas for others there may be no difference (Campbell and Green, 1965). The panel reached consensus (82%) that classification should be based on the results when testing both eyes together rather than the current practice of classification using the test results of the best eye only.

Classification is currently conducted at a competition venue or a local optometry/ophthalmology clinic prior to the start of competition. In our initial survey, some panelists raised concerns about this approach due to: (i) differences in testing conditions across the different competition events; (ii) not enough time being available for classifiers to fully examine visual function during classification prior to a competition, leaving classifiers to rely on medical documents provided by the athletes which are of various standards. One potential solution raised by panelists was the establishment of “classification centers” where athlete evaluation would be conducted, rather than classifiers traveling to competition venues. At these centers, both the medical assessment to establish the athlete’s medical condition, as well as the tests of visual function could be conducted. All athletes need to be classified prior to taking part in competition and depending on the stability of their medical condition, their classification needs to be reviewed every year, 2 years, 4 years, or is confirmed. The panel reached consensus (82%) that they would favor the use of centralized classification centers over the current method of classification at competition. The main consideration was that classification centers would increase the quality and credibility of classification, although some panelists objected because of the additional time and (financial) resources required to travel to these centers.

### Section 7: Impact of Vision Impairment Across Different Weight Categories

The same classification criteria apply to all judokas irrespective of their weight category, although it remains unknown whether the impact of VI is identical across all weight categories. The time constraints in combat sports as well as the techniques and tactics used have been shown to differ across weight categories (Franchini et al., 2011; Miarka et al., 2015; Tabben et al., 2015). These different task constraints imposed by an athlete’s weight may require different perceptual abilities. We raised this topic with our panel, but reached no consensus whether the impact of VI differs across weight categories. About half (56%) of the panelists believed that the impact of VI is greater in the lighter weight-categories, largely because competition requires more and faster movements than matches in heavier weight categories. However, the panel clearly agreed (93%) that the impact on performance does *not* vary enough across weight categories to warrant the consideration of unique classification criteria for different weight categories. Some panelists commented that there may be subtle differences, but these would not warrant separate classification criteria. Others commented that separate criteria for different weight categories would render classification overly complex.

### Section 8: Impact of a Congenital Versus Acquired Vision Impairment

VI classification currently does not take into account the age at which an athlete acquires their VI, even though this might have a considerable impact on their ability to acquire sport-specific skill (Ravensbergen et al., 2016). The panel reached consensus (79%) that in judo, the impact of impairment on performance is likely to be influenced by the age at which VI is acquired. The main comment was that partially sighted athletes might hold an advantage in skill learning over those who learn without vision (by making use of observational learning). Conversely though, some panelists noted that athletes with congenital impairments might be better able to use other sensory information (i.e., haptic, kinesthetic) than athletes with acquired impairment. Nonetheless, the majority of panelists (62%) believed that a judoka with acquired impairment has an advantage over a judoka with congenital impairment, with the remaining 38% answering that the impact on performance is the same for all judokas. Panelists commented that the answer to this question depends not only on the age at which the impairment was acquired, but also on the age at which the athlete was introduced to judo, and to individual differences in learning styles. The panel did agree (89%) that the age at which an impairment is acquired should *not* be taken into account for classification in VI judo. Their main comment was that the issue would be too complex to accurately account for in classification.

### Section 9: The Use of Blindfolds

A seemingly straightforward method to standardize the level of impairment during competition would be to require all judokas to compete blindfolded; however, blindfolding is generally not considered an acceptable solution for most in the VI sports community (Mann and Ravensbergen, 2018). First there are ethical concerns about blindfolding, because partially sighted athletes become even further impaired. Moreover, blindfolds do not account for advantages afforded to partially sighted athletes away from competition (e.g., in training). Nevertheless, according to experts in VI sports, there may be specific situations in which the use of blindfolds is warranted (Ravensbergen et al., 2016). We therefore discussed with our panel the potential suitability of blindfolds for use within VI judo. The panel did not agree whether the use of blindfolds would provide an *appropriate* way to create fair competition in VI judo (31% yes; 69% no). In a follow-up question, the panel reached consensus (94%) that blindfolding all competitors would *not decrease* the fairness of VI judo: 65% believed that blindfolds would make VI judo fairer than it currently is; 29% believed that it would not impact the fairness; 6% believed that blindfolds would make VI judo less fair. Still, the panelists could not agree whether they would be *in favor* of the use of blindfolds in VI judo (33% yes; 67% no). Some additional concerns in terms of implementation were that the use of blindfolds might be too dangerous, because partially sighted athletes might presently rely on their vision to fall safely, or too impractical, because blindfolds might not stay on during competition. However, the panel did not agree with these concerns (too dangerous: 38% yes, 64% no; too impractical: 71% yes, 29% no). In conclusion, no consensus was reached here, although the majority of the expert panel expressed that they would not support the introduction of blindfolds in VI judo.

### Section 10: Intentional Misrepresentation

Athletes who deliberately underperform on classification tests are guilty of the *intentional misrepresentation* (IM) of their abilities. The IPC considers IM to be a serious offense because it represents a significant threat to the legitimacy of Paralympic competition (International Paralympic Committee, 2015). However, even though strong penalties are in place, detecting IM represents a challenge, because very few objective measures are available to detect whether or not athletes provided honest answers on vision tests used during classification (Mann and Ravensbergen, 2018). The panel reached consensus (94%) that there currently are some VI judokas who intentionally misrepresent their visual abilities during classification. In a follow-up question, 88% of the panelists answered they believe there are some VI judokas currently competing who should actually be classified as not eligible, but have been classified eligible to compete because they intentionally misrepresented their visual abilities.

We then asked our panel to identify measures that might help to minimize IM, and to rate how effective each approach would be (see Table 6). The panel agreed that the introduction of less subjective testing methods would be “very” or “extremely” effective (94%). Other approaches rated as “very” to “extremely” effective were: out-of-competition testing (i.e., unexpected visits of classifiers to athletes to conduct classification tests), the observation of athlete behavior (e.g., in training or competition), and stricter requirements for the medical documentation of the athlete’s vision condition submitted prior to classification. Measures rated at least moderately effective were: holding national federations accountable for an athlete’s IM (currently, only athletes themselves are held responsible), the possibility to express doubts about the outcome of classification for individual athletes, and the introduction of centralized classification centers. The only measure for which the panel showed low agreement was the requirement for all athletes to wear blindfolds, with about half of the panel rating this as “not at all” to “slightly” effective (47%) and the other half (53%) rating it “moderately” to “extremely” effective.

**Table 6:** Relative effectiveness of measures to reduce the incidence of intentional misrepresentation in VI judo.

Measures to reduce the incidence of intentional misrepresentation	Not at all effective	Slightly effective	Moderately effective	Very effective	Extremely effective	Level of consensus for effectiveness to reduce intentional misrepresentation
Introduce less subjective testing methods during classification	0%	0%	6%	44%	50%	Very to extremely (94%)
Incorporate out-of-competition testing	0%	18%	6%	35%	41%	Very to extremely (76%)
Include observation in classification	6%	12%	6%	35%	41%	Very to extremely (76%)
Introduce stricter requirements for medical diagnostic forms	0%	6%	19%	31%	41%	Very to extremely (75%)
Introduce centralized classification centers	0%	6%	25%	25%	44%	Moderately to extremely (94%)
Hold national federations accountable for an athlete’s IM	6%	6%	19%	31%	38%	Moderately to extremely (88%)
Introduce the possibility to file doubts about an athlete’s classification	6%	13%	31%	41%	6%	Moderately to extremely (81%)
Require all athletes to use blindfolds/eyeshades during competition	35%	12%	12%	18%	24%	Not at all to very (76%)


*Panelists were asked to answer on a five-point Likert scale ranging from “not at all effective” to “extremely effective.” The level of consensus was determined by taking the smallest range of answers given by at least 75% of the panelists. The list is ordered according to the rankings of the experts from most to least effective.*

## Discussion

Providing legitimate competition for all athletes is at the heart of the Paralympic movement. Classification is therefore considered to be “the sole means by which success in Paralympic sport is legitimized” (Tweedy and Howe, 2011, p. 19). The aim of this study was to gather expert opinions to guide the development of an evidence-based, sport-specific classification system for VI judo. We found consensus that the current classification system does not fully achieve its aim of minimizing the impact of impairment on the outcome of competition. The findings highlight the need to change the way classification is structured for VI judo, and provide clear guidance for how the relationship between impairment and performance can be examined to develop judo-specific classification. Specifically, the expert panel provided guidance on (i) which aspects of vision are most likely to impact judo performance, (ii) which aspects of judo performance are most likely to be impacted by VI, (iii) under what conditions vision testing for judo classification should take place, and (iv) other issues specific to classification research in VI judo. These findings help to design experimental studies to test the association between relevant measures of VI and measures of judo performance. They also provide theoretical underpinning for how VI limits judo performance.

### Measures of Visual Function

The current results provide guidance for how to best conduct empirical research to determine the impairment–performance relationship in VI judo. A critical step in sport-specific classification research is to select accurate and reliable measures of impairment that are likely to impact performance in that sport (Tweedy et al., 2016). Our expert panel identified, in addition to the current measures (i.e., visual acuity and visual field), six aspects of vision (motion perception, dynamic visual acuity, light sensitivity, ocular coordination, depth perception, and contrast sensitivity) that should be incorporated in empirical research to establish which are related to judo performance and therefore should be tested during classification. It is possible that other aspects of visual function are more predictive of judo performance than those measures currently tested. For instance, contrast sensitivity has proven to be more predictive for performance in VI shooting than visual acuity (Allen et al., 2018).

### Analysis of Judo Performance

The expert consultation also provided guidance for how best to analyze and measure judo performance. Our panelists identified six aspects of performance that they considered to be both impacted by VI and are decisive for winning a contest: grip fighting skills, offensive skills in the standing fight, defensive skills in the standing fight, transition from standing to ground fight, offensive skills in the ground fight, and defensive skills in the ground fight. Together these cover a broad range of judo skill, and empirical research should thus preferably take an equally broad perspective to performance. This might be achieved by considering the individual aspects separately, i.e., by counting the number of scores obtained in the ground fight, or calculating the percentage of successfully defended attacks in a standing fight. However, many elements of judo performance interact with each other and are therefore not independent: obtaining a slightly advantageous grip provides additional possibilities to attack, and the way an attack in the standing fight is executed largely determines the course of events during the following ground fight. Overall determinants of judo performance which bring together these measures may also be included in research to appraise performance, incorporating measures of match outcome, score count, or percentage of matches won in a tournament.

### Vision Testing Procedures

Wherever possible, VI classification should be conducted using the same conditions as those experienced during competition (Mann and Ravensbergen, 2018). Classification outcomes are currently determined using the test results of the best eye only, whereas in competition athletes would normally rely on both eyes. Also, in classification testing is done with the best possible optical correction, irrespective of whether or not that correction can reasonably be worn on the mat in competition. In agreement with the VI position stand, our panel reached consensus that classification should be based on results from vision tests conducted while using *both eyes*, because this represents the habitual function used during competition. Moreover, the panel remained of the opinion that classification should continue to be based on test results wearing the best possible optical correction, rather than on the level of vision that athletes have available when competing, largely because athletes might still benefit from better vision away from competition (i.e., in training).

### Confounding Factors

The expert panel reached consensus that both the weight category of the judoka and the age at which they acquired their impairment should not be considered in classification, even though there was some suggestion that both mediate the impairment–performance relationship in judo. The panelists felt that an effort to address these matters would make classification overly complex. However, complexity is not necessarily a good justification for avoiding these issues. The VI position stand therefore encourages those involved in classification research to collect data on the developmental history of the impairment in combination with their developmental history within the sport wherever possible, so that the impact of impairment on performance could be compared between athletes who have similar impairments but developed at different ages. Likewise, it would be advisable to determine whether there is an interaction between the weight category of an athlete and the nature of the impairment–performance relationship to test whether the scientific evidence supports the views of our panel.

### Intentional Misrepresentation

The expert panel expressed serious concerns about the threat of IM to the fairness of competition, which is an issue of broader concern across VI sports (Ravensbergen et al., 2016). Because VI judo presently implements only one sport class during competition, there is little advantage for a truly eligible athlete to be allocated to a class designed for athletes with more severe impairments beyond the provision of more points for the purpose of qualification. The key concern of the panel appears to be for athletes who should not meet the MIC, but misrepresent their ability to become eligible to compete. Although the primary aim of classification research is to evaluate the suitability of the sport class criteria rather than the fairness of the athletes competing within those classes, IM offers a serious threat to the legitimacy of VI sports. It would therefore seem worthwhile for both classification researchers and governing bodies of VI sports to consider measures that would prevent the incidence of IM and increase the trust of the VI sports community in their classification systems. The highest priority of our panel was to introduce less subjective testing, because current methods rely on athletes to provide their best effort and honest answers. One approach to increase objectivity in classification is to take into account the consistency of test performance, which may hold promise as an indicator whether or not the athlete provided their best effort and honest answers (Deuble et al., 2016; Ravensbergen et al., 2018).

## Conclusion

According to our expert panel, changes are needed to the manner in which classification is conducted in VI judo. The panelists call for a re-evaluation of the minimum level of VI needed to qualify to compete, and they question the idea that all VI judokas can compete against each other equitably within a single sport class. The expert panel helped us to identify which aspects of VI are most likely to impact judo performance, and what aspects of judo performance are most likely to be impacted by VI. The Delphi method proved to be a useful tool to gather expert opinions as a starting point for the development of sport-specific classification for VI judo. The current findings will guide future empirical research from which new, evidence-based classification criteria for VI judo can be established. The ultimate aim of this research is to improve the fairness of VI judo competition, and to ensure that Paralympic medals awarded in judo are, as US Olympian Dan Gable famously quoted, made of “sweat, determination, and a hard-to-find alloy called guts.”

## Data Availability

Responses on final questions posed in each topic can be found in Supplementary Table S1.

## Ethics Statement

The study was approved by the Scientific and Ethical Review Board of the Faculty of Behavioural and Movement Sciences at the Vrije Universiteit Amsterdam. All participants provided electronic informed consent to take part in accordance with the Declaration of Helsinki.

## Author Contributions

All authors conceived and designed the study. KK distributed the surveys and collected responses. Between rounds, all authors discussed responses and designed follow-up surveys. HN also translated surveys and responses for one Japanese panelist. All authors wrote and approved the final manuscript.

## Conflict of Interest Statement

The authors declare that the research was conducted in the absence of any commercial or financial relationships that could be construed as a potential conflict of interest.

## Footnotes

1 Some discussion has been going on whether an athlete should be allowed to compete simultaneously in Olympic and Paralympic competition, especially sparked by Oscar Pistorius who was the first amputee to compete in both the Paralympic and Olympic Games (e.g., van Hilvoorde and Landeweerd, 2008; Wolbring, 2012).

2 Athletes can be classified to compete based on test of either visual acuity, a measure of the sharpness of their vision, or visual field, a measure of the area of peripheral vision with which they can see. To be classified as B3, an athlete needs to have a visual acuity between 1.0 and 1.4 logMAR, or a visual field restricted to less than 40° diameter. To be classified as B2, an athlete needs to have a visual acuity between 1.4 and 2.6 logMAR, or a visual field restricted to less than 10° diameter. Athletes with visual acuity of 2.6 logMAR or worse are classified as B1.

3 Currently, judokas earn progressively more points if they win against an opponent from a vision class designed for athletes with less severe impairment, e.g., when a B1 judoka fights a B3 judoka, the B1 judoka would earn 50 ranking points for winning but only 30 points if the opponent is in the B1 class. See http://www.ibsasport.org/sports/files/387-Rules-IBSA-Judo-world-ranking-list—points-system.pdf.

4 One coach was unable to complete the final survey due to personal circumstances.

5 Visual acuity is a measure of the sharpness/clarity of vision. Vision that is 6/6 or 20/20 equals logMAR 0.0, vision that is 6/60 or 20/200 (sometimes loosely referred to as “10% vision”) is logMAR 1.0.

6 Visual field is a measure of the area of peripheral vision with which an individual can see (i.e., without moving their eyes).

7 We provided the panelists with the opportunity to add other visual functions, to which an impairment might lead to a decrease in judo performance, to our proposed list. None of the panelists did so, indicating that all visual functions they believed might impact judo performance were included.
