# Supplementary material for: The Development of Evidence-Based Classification of Vision Impairment in Judo: A Delphi Study
Source: Front Psychol. 2019 Feb 15;10:98. doi: 10.3389/fpsyg.2019.00098 (PMC6384233; doi:10.3389/fpsyg.2019.00098)
Supplement: Supplementary file 1 [file Table_1.DOCX]

Supplementary Table 1. Final questions posed in each of the 10 sections of the survey.

| Section | Questions | Answers: # (*% after exclusion*) | Consensus? |
| --- | --- | --- | --- |
| 1. Aim of classification | Do you believe that the way that vision impairment is currently classified within VI judo fulfils the aim to minimise the impact of vision impairment on the outcome of competition? | Yes: **3 (17*%*)**  No: **11 (61*%*)**  Partially: **4 (22*%*)**  I don’t feel qualified: **0** | Yes (that the aim is not entirely fulfilled) – after round 1 |
| 2. Minimum impairment criteria | The minimum impairment criteria for VI judo should be: | Less inclusive, excluding some athletes who are currently eligible to compete: **8 (62*%*)**  More inclusive, allowing some athletes to compete who currently do not qualify to take part: **0 (0*%*)**  Stay as it is: **5 (*38%*)**  I don’t feel qualified: **5** | Yes (that it should not be more exclusive) – after round 2 |
|  | Do you believe that further research is needed to define the minimum level of vision impairment that would decrease performance in judo? | Yes: **12 (*80%*)**  No: **3 (*20%*)**  I don’t feel qualified: **3** | Yes – after round 2 |
|  | Imagine an athlete who is eligible to compete in VI judo, but wants to compete in able-sighted judo as well. Do you believe an athlete should be allowed to compete in both able-sighted judo and in VI judo? | Yes: **15 (*83%*)**  No: **3 (17*%*)**  I don’t feel qualified: **0** | Yes – after round 1 |
|  | Do you believe that it is possible for some athletes with vision impairment to be eligible to compete in VI judo, yet they may be so skilled and/or so well trained that they are still able to compete in judo for able-sighted athletes at the elite level (e.g., in World Championships or in the Olympic Games)? | Yes: **8 (44*%*)**  No: **10 (56*%*)**  I don’t feel qualified: **0** | No |
| 3. Sport classes | Do you believe that the current point system helps judokas with more severe vision impairment to qualify for the Paralympic  Games? | Yes: **13 (*81%*)**  No: **3 (*19%*)**  I don’t feel qualified: **1** | Yes – after round 3 |
|  | Within the current competition format, where all VI judokas compete together, which of these systems do you believe to be the fairest to use for Paralympic qualification? | A point system for qualification: **14 (*82%*)**  No point system for qualification: **3 (*18%*)**  I don’t feel qualified: **0** | Yes – after round 3 |
|  | Do you believe that splitting VI judo into more than one sport class would create a more fair competition? | Yes: **14 (*78%*)**  No: **4 (*22%*)**  I don’t feel qualified: **0** | Yes – after round 1 |
|  | Disregarding any practical consequences (e.g. a lower number of competitors per class), do you believe that having a separate class for judokas who are completely blind would be fairer than the current competition format where all VI judokas compete against each other? | Yes: **14 (*78%*)**  No: **4 (*22%*)**  I don’t feel qualified: **0** | Yes – after round 2 |
|  | Which of these proposals would you like most:  - Proposal 1, where there would be two VI classes (i.e. for blind and partially sighted athletes) that compete separately, and a reduction in the number of weight categories to maintain the same number of medal events.  - Proposal 2: where there would be two VI classes (i.e. for blind and partially sighted athletes) that compete separately, and the current weight classes are maintained, but only the events for blind judokas would be on the program for the Paralympic Games.  - The present system, where all athletes compete together. | Proposal 1: **5 (*38%*)**  Proposal 2: **4 (*6%*)**  The present system: **7 (*6%*)**  I don’t feel qualified **1** | No |
| 4. Measures of visual function to be used during classification | Do you believe that the assessment of visual acuity and visual field are the only measures of visual function that should be used for classification in VI judo? | Yes: **5 (*42%*)**  No: **7 (*58%*)**  I don’t feel qualified: **6** | No |
|  | Please indicate which of the following aspects of visual function you believe to be important enough to include in classification for VI judo. | See Table 3 in text | See Table 3 in text |
| 5. Impact of vision impairment on different aspects of performance | For the following aspects of judo performance, please indicate how important you believe they are for winning a match in VI judo. | See Table 4 in text | See Table 4 in text |
|  | Please indicate for each of these specific aspects of performance to what extent you believe that fully sighted judokas would on average hold an advantage when competing against judokas who just meet the current minimum impairment criteria to be eligible to compete in VI judo (of similar fighting experience), when fighting under VI judo rules. | See Table 5 in text | See Table 5 in text |
|  | Please indicate for each of these specific aspects of performance to what extent you believe that fully sighted judokas would on average hold an advantage when competing against blind judokas (of similar fighting experience), when fighting under VI judo rules. | See Table 5 in text | See Table 5 in text |
| 6. Vision testing conditions | Classification should be based on test results from: | The best eye only: **3 (*18%*)**  Both eyes together: **14 (*82%*)**  I don’t feel qualified **1** | Yes – after round 2 |
|  | Classification should be based on the tests of when the athlete wears: | Their best possible optical correction irrespective of whether it can be worn during competition: **13 (*76%*)**  Their best possible optical correction that could be worn during competition: **3 (*18%*)**  Without any optical correction: **1 (6*%*)**  I don’t feel qualified **1** | Yes – after round 1 |
|  | Would you be in favour of the use of centralised classification centres instead of  classification being held at competition venues? | Yes: **14 (*82%*)**  No: **3 (*18%*)**  I don’t feel qualified: **1** | Yes – after round 2 |
| 7. Impact of vision impairment across different weight categories | Do you believe that vision impairment is more likely to impact the performance of lightweight judokas than it would for heavyweight judokas? | Yes: **9 (*56%*)**  No: **7 (*44%*)**  I don’t feel qualified: **1** | No |
|  | Do you believe that the impact of vision impairment on performance varies across the weight classes so much, that VI classification criteria should be considered for each weight category independently? | Yes: **1 (*7%*)**  No: **14 (*93%*)**  I don’t feel qualified: **2** | Yes – after round 3 |
| 8. Impact of a congenital compared to an acquired impairment | For a judoka with vision impairment, do you believe that the age at which the impairment was acquired influences the impact of the impairment on judo performance during competition? | Yes: **13 (7*6%*)**  No: **4 (2*4%*)**  I don’t feel qualified: **1** | Yes – after round 1 |
|  | Do you believe that the age at which a vision impairment is acquired should be taken into account during classification? | Yes: **2 (*11%*)**  No: **16 (*89%*)**  I don’t feel qualified: **0** | Yes – after round 1 |
| 9. The use of blindfolds | Do you believe that the use of blindfolds would be an appropriate way to create fair  competition in VI judo? | Yes: **5 (*31%*)**  No: **11 (*69%*)**  I don’t feel qualified: **2** | No |
|  | Requiring all VI judokas to wear blindfolds/eyeshades during competition would: | Make competition fairer than it currently is: **11 (*65%*)**  Not impact the fairness of competition: **5 (*29%*)**  Make competition less fair than it currently is: **1 (*6%*)** | Yes (that it will not make competition less fair) – after round 3 |
|  | Would you be in favour of the use of blindfolds / eyeshades during VI judo  competition? | Yes: **6 (*33%*)**  No: **12 (*67%*)**  I don’t feel qualified: **0** | No |
|  | Do you believe that requiring all athletes to wear blindfolds/eyeshades would be too  dangerous in VI judo competition? | Yes: **5 (*36%*)**  No: **9** **(*64%*)**  I don’t feel qualified **3** | No |
|  | Do you believe that requiring all athletes to wear blindfolds/eyeshades would be too  unpractical in VI judo competition (i.e., they are likely to fall off during competition). | Yes: **12 (*71%*)**  No: **5** **(*29%*)**  I don’t feel qualified: **0** | No |
| 10. Intentional misrepresentation | Do you believe that some VI judo athletes are currently intentionally misrepresenting their level of visual ability during classification? | Yes: **15 (*94%*)**  No: **1 (*6%*)**  I don’t feel qualified. **2** | Yes – after round 1 |
|  | Do you believe that there are some VI judo athletes currently competing who should actually be classified as not eligible, but have been found eligible to compete because they intentionally misrepresented their level of visual ability? | Yes: **14 (*93%*)**  No: **1 (*7%*)**  I don’t feel qualified. **0** | Yes – after round 1 |
|  | Please indicate how effective you believe that each of the following approaches would be in helping to minimise the incidence of intentional misrepresentation during classification. | See Table 6 in text | See Table 6 in text |
