# Supplementary material for: The Development of Evidence-Based Classification of Vision Impairment in Judo: A Delphi Study
Source: Front Psychol. 2019 Feb 15;10:98. doi: 10.3389/fpsyg.2019.00098 (PMC6384233; doi:10.3389/fpsyg.2019.00098)
Supplement: Supplementary file 2 [file Table_2.DOCX]

Supplementary Table 2. Process of reaching consensus on the central questions posed in each of the 10 sections of the survey.

| Section | Central question(s) | Result/action after survey 1 | Result/action after survey 2 | Result after survey 3 |
| --- | --- | --- | --- | --- |
| 1. Aim of classification | Does the current system of classification within VI judo achieve its aim of minimising the impact of impairment on the outcome of competition? | **Consensus reached** |  |  |
| 2. Minimum impairment criteria | Are the current minimum impairment criteria for VI judo set appropriately or should they change, and if so how? | Question rephrased | **Consensus reached** |  |
|  | Do you believe an athlete should be allowed to compete in both able-sighted judo and in VI judo? | **Consensus reached** |  |  |
| 3. Sport classes | Is it appropriate that all eligible athletes in VI judo compete against each other, or should additional sport classes be created? | **General consensus reached**  Concerns addressed and further exploration for creating a separate sports class for blind athletes | **Consensus reached** |  |
|  | Do you believe that it is currently necessary to have a points system, where the number of points earned is dependent on the level of impairment of the judoka and their opponent? | Question rephrased | Question rephrased | **Consensus reached** |
| 4. Measures of visual function to be used during classification | Which aspects of vision are most likely to impact on judo performance? | List of aspects of visual functions confirmed | Rating of perceived importance of each visual function to be included in classification. |  |
| 5. Impact of vision impairment on different aspects of performance | Which aspects of performance are most likely to be impacted by vision impairment? | List of performance aspects put together which might be impacted by vision impairment. | Question rephrased | Ratings of impact of VI and importance for winning for all performance aspects. |
| 6. Vision testing conditions | Should classification be based on the results obtained wearing the best possible optical correction? | **Consensus reached** |  |  |
|  | Should classification be based on the results of the best eye? | Question clarified | **Consensus reached** |  |
| 7. Impact of vision impairment across different weight categories | Does the impact of vision impairment on judo performance differ across weight categories? | Concerns addressed | Question rephrased | No consensus reached |
|  | Do you believe that VI classification criteria should be considered for each weight category independently? |  | Concerns addressed | **Consensus reached** |
| 8. Impact of a congenital compared to an acquired impairment | Does the impact of vision impairment on judo performance differ between athletes with congenital and acquired impairments? | **Consensus reached** |  |  |
|  | Do you believe that the age at which a vision impairment is acquired should be  taken into account during classification? | **Consensus reached** |  |  |
| 9. The use of blindfolds | Would the use of blindfolds be an appropriate way to ensure fair competition within VI judo? | Question rephrased | Question rephrased | Partial consensus reached |
| 10. Intentional misrepresentation | Do you believe that some VI judo athletes are currently intentionally misrepresenting their level of visual ability during classification? | **Consensus reached**  Additional question posed on preventive measures against IM | List of preventive measures against IM put together | Rating of perceived effectiveness of preventive measures against IM |
